# 

**Published:** 1872-02

**Authors:** 


### TABLE OF CONTENTS.

ORIGINAL COMMUNICATIONS:

SENILE AND TRAUMATIC CATARACT. A Clinical Lecture Delivered at the University Medical College, New York, December 20, 1871. By D. B. ST. JOHN ROOSA, M.D., Clinical Professor of Diseases of the Eye and Ear in the University, and Surgeon to the Manhattan Eye and Ear Hospital, New York.......................... 611

SUSPENDED FŒTAL ANIMATION. By A. W. GRIGGS, M.D., West-Point, Ga...616

CONGESTIVE CHILL, ASSOCIATED WITH HÆMATURIA—BOTH DEPENDENT UPON MALARIAL INFLUENCE. By W. O'DANIEL, M.D., Twiggs County, Ga., Treasurer of the Georgia Medical Association ...................... 619

ATLANTA ACADEMY OF MEDICINE. J. T. JOHNSON, M.D., Reporter....... 622

ABSTRACTS FROM GERMAN JOURNALS. By CH. RAUSHENBERG, M.D., Atlanta 634

(Croup and Diphtheritis. By Dr. Franz Hartman, at Wiesbaden. Virchow's Archiv., February, 1871.——Diphtheritis and Diphtheria. By Dr. L. Letzerich, Idstein, near Wiesbaden.)

SELECTED ARTICLES:

CLINICAL LECTURE ON CONJUGAL ONANISM AND KINDRED SINS. By WILLIAM GOODELL, M.D., Clinical Lecturer on Diseases of Women and Children in the University of Pennsylvania................................ 644

PHYSIOLOGICAL ACTION OF CHLORAL HYDRATE. By M. A. BYASSON....... 652

TREATMENT OF PRURITUS VULVÆ.......................................... 653

OPHTHALMIA NEONATORUM. A Clinical Lecture at the Dispensary of the Medical College of Ohio. By Prof. SEELY.......................... 654

LIQ. MAGNESIÆ—A REMEDY FOR CARDIALGIA (HEARTBURN.)............ 657

EDITORIAL AND MISCELLANEOUS:

THE MUTUAL RELATIONS OF THE MEDICAL PROFESSION AND ITS PRESS...... 659

ATLANTA ACADEMY OF MEDICINE........................................ 660

CRIMINAL ABORTION.................................................. 661

INEBRIATE ASYLUM.——DR. R. A. T. RIDLEY.......................... 662

A STEP IN THE RIGHT DIRECTION.——BOOKS RECEIVED.................. 663

MIDDLE GEORGIA MEDICAL SOCIETY..................................... 664

TO MEDICAL PUBLISHERS, MEDICAL ASSOCIATIONS, AND PHYSICIANS ....... 665

MISCELLANEOUS...................................................... 666

(Death of Dr. R. A. T. Ridley.——Diagnosis of Syphilis by the Microscopic Examination of the Blood.——A Few Words About Homœopathy.——Diagnosis of Phantom Abdominal Tumors.)

